# A Theoretical Analysis of the Differential Chemical Reaction Results Caused by Chirality Induction

**DOI:** 10.3390/molecules28176286

**Published:** 2023-08-28

**Authors:** Feng-Yu Zhang, Sicheng Liu, Anwei Huang, Yi-Ning Li, Xiao-Yan Liu, Peng Zhang

**Affiliations:** 1 School of Space Science and Physics, Shandong University, Weihai 264209, China; fengyuzhang@mail.sdu.edu.cn (F.-Y.Z.); liusicheng@mail.sdu.edu.cn (S.L.); yiningli@mail.sdu.edu.cn (Y.-N.L.); liuxiaoyan@mail.sdu.edu.cn (X.-Y.L.); 2 School of Basic Medicine, Shanxi Medical University, Jinzhong 030600, China; huanganwei@sxmu.edu.cn

**Keywords:** chiral drugs, chirality-induced spin selectivity (CISS), chiral enzymes, L-DOPA, AADC enzyme

## Abstract

The theory of electron spin has been proposed for a century, but the study of quantum effects in biological molecules is still in its infancy. Chirality-induced spin selectivity (CISS) is a very modern theory that can explain many biochemical phenomena. In this paper, we propose a new theoretical model based on CISS theory and quantum chemistry theory, which can well explain the theoretical explanation of the chiral selectivity of chiral proteins. Moreover, this theory can predict the spin state of corresponding chiral molecules. Taking the L-DOPA and AADC enzymes as examples, this theoretical model elucidates the AADC enzyme’s chiral catalysis selectivity and successfully predicts the spin state of L-DOPA and D-DOPA’s valence electrons.

## 1. Introduction

Research on chiral molecules has forged ahead since their discovery. In 1848, the concept of chiral molecules was first proposed by Louis Pasteur, who made the initial observation of optical rotation in substances. In recent years, as research on chiral drugs has deepened, an increasing number of chiral drugs have entered clinical research and been brought to the market, including moxifloxacin and levofloxacin, among others. Meanwhile, there is a growing focus on issues such as the formulation, safety evaluation, and pharmacological efficacy assessment of chiral drugs.

Due to the chirality of the majority of drug molecules, different enantiomers of drug molecules may have distinct effects on the human body. Therefore, the synthesis of single enantiomers in drug molecules is of paramount importance in pharmaceutical development. In 1990, Elias J. Corey was awarded the Nobel Prize in Chemistry for his contributions to asymmetric synthesis. He introduced a novel organic synthesis method that involves designing and combining simple molecular units to achieve the asymmetric synthesis of complex molecules. This research provided new insights and approaches for the study of chiral drugs [1]. In 2001, William S. Knowles, Ryoji Noyori, and K. Barry Sharpless were jointly awarded the Nobel Prize in Chemistry for their pioneering work on chiral catalysts. Notably, Ryoji Noyori authored a paper titled “The Asymmetric Hydrogenation of β-Keto Carboxylic Esters Catalyzed by Chiral Ruthenium Complexes.” This study delved into the application of chiral catalysts in asymmetric hydrogenation reactions for synthesizing chiral drugs. Noyori’s work unveiled that chiral catalysts effectively drive asymmetric hydrogenations, yielding products with exceptional enantioselectivity and high yields. His research thus laid a vital foundation and theoretical framework for chiral drug synthesis [2]. In biological systems, chirality abounds in biomolecules. Most notably, levorotatory amino acids constitute the bulk of proteins, forming peptide chains that intricately fold to create proteins with dominant levorotatory structures. However, the fundamental rationale behind nature’s resolute preference for chirality remains enigmatic, despite the energetic expenses associated with upholding this symmetry. Quantum mechanics’ rapid evolution in the 1920s has spurred the growth of various interdisciplinary fields. The unearthing of the CISS (Charge-Induced Spin Selectivity) [3] effect has catalyzed the development of organic devices grounded in electron spin manipulation.

At Israel’s Weizmann Institute of Science, Ron Naaman embarked on unraveling the intricate relationship between electron spin, molecular chirality, and their profound implications for vital biological processes. He harnessed the CISS effect as a cornerstone for his investigations. Naaman’s exploration underscored the pivotal role of chiral molecular rotation in diverse biological processes, such as protein folding and enzymatic catalysis. Additionally, he discussed advancements in leveraging chiral molecular spin to govern electron transport and explored the potential applications of these insights within the realms of biology and medicine [4].

Building upon the CISS theory, this paper proposes a theoretical explanation for the differential effects produced by drug molecules of different enantiomers on the human body. Theoretical calculations and explanatory discussions are conducted using L-DOPA and D-DOPA as examples, targeting the AADC enzyme. Finally, the relationship between optical rotation and the electron spin of chiral substances is briefly discussed.

## 2. Chiral-Induced Spin Selectivity (CISS)

The essence of a chemical reaction lies in the transfer of electrons, leading to the formation or breaking of atomic bonds. In classical chemical theory, the process of a chemical reaction is solely based on the consideration of electron charge. However, in modern quantum chemical theory, factors such as the wave-like nature of electrons and electron spin are also taken into account, providing a more accurate and comprehensive explanation of chemical reactions.

**Figure 1:**
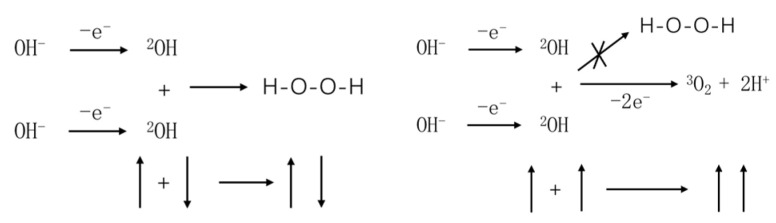
Reaction pathways of the OH radical in different spin states (The upward arrow indicates electron spin-up; the downward arrow signifies electron spin-down).

As shown in Figure 1, two OH radicals undergo a chemical reaction. When the spins of the valence electrons on the two OH radicals are antiparallel, these radicals tend to form hydrogen peroxide molecules, resulting in covalent bond formation. The reaction outcome is depicted in the left diagram. Conversely, when the spins of the valence electrons on the two OH radicals are parallel, the total orbital wave function must be antisymmetric under exchange, meaning that these two valence electrons must couple in different orbitals, due to the fact that only spin-coupled wave functions such as $\kappa_{1\;1}$ and $\kappa_{1-1}$, which are symmetric under exchange, can be formed by the coupling of these valence electrons. Consequently, the reaction occurs on the triplet state surface. The formed chemical bond would be highly unstable, rapidly leading to the formation of molecular oxygen. The reaction outcome is illustrated in the right diagram. This result has been previously confirmed by Mtangi et al. in experimental studies [5].

In 1990, German physicists conducted an experiment where they emitted electrons toward the volatile odor molecules (a type of protein) of a camphor tree. They observed a slight bias in the spin of electrons passing through the molecules: electrons with spin pointing upward seemed to preferentially pass through left-handed chiral molecules, while electrons with spin pointing downward showed a preference for right-handed ones. This phenomenon is now known as “chiral-induced spin selectivity (CISS)”.

The CISS effect has garnered sustained attention within the scientific community. Although its initial observation dates back to 1990, it was not until 2013 that comprehensive and systematic investigations began [6]. In 2014, researchers discerned its connection to the relative orientation of chiral molecules, specifically manifesting in molecules with a particular degree of symmetry [7]. Recent years have brought to light that the CISS effect extends to more intricate molecules and can be modulated by adjusting molecular structures, holding notable implications for innovative chiral molecular material design [8]. Moreover, scholars have unveiled the CISS phenomenon’s manifold impacts on biological systems. It has the potential to expedite long-distance electron transfer, augment biological affinity and enantioselectivity, and facilitate efficient and selective multi-electron oxidation-reduction processes [9]. Leveraging the insights from the CISS effect and the findings presented above, it becomes evident that chiral molecules can strategically influence participation in specific reactions by manipulating electron spins. This perspective may offer insights into the intricacies of biological recognition reactions [10].

Various theoretical assumptions have emerged in attempts to elucidate the underlying mechanisms of the CISS effect. One theory proposes that chiral molecules serve as spin polarizers, influencing the polarization of all scattered electrons (whether transmitted or reflected) in a consistent direction. This direction hinges on both the molecule’s chirality and the angle of electron incidence [11]. Researchers have explored the correlation between chiral-dependent spin polarization and the imaginary component of the effective single-electron Hamiltonian matrix, particularly for heavy-atom molecules [12]. Moreover, the utilization of helical structures as spin filters to achieve polarization, independently of an external magnetic field, has been highlighted [13]. Certain scholars have substantiated these hypotheses through quantum transport calculations within the framework of density functional theory, offering quantitative insights at a single-particle level [14]. However, a theoretical consensus has yet to be established [15]. Regardless of the prevailing theory, this effect’s significance persists in propelling the applications of chiral molecules across domains like life sciences and nanoelectronics.

## 3. Results

### 3.1. Electron Spin and Chemical Reactions

The concept of electron spin was first introduced by physicists Samuel Goudsmit and George Uhlenbeck in their paper published in 1925. The paper proposed that electrons possess not only charge and mass but also intrinsic angular momentum [16]. Similar to electron charge, electron spin is an inherent property of electrons, dictating the behavior of electrons in external magnetic fields and influencing the properties and reactions of matter.

**Figure 2:**
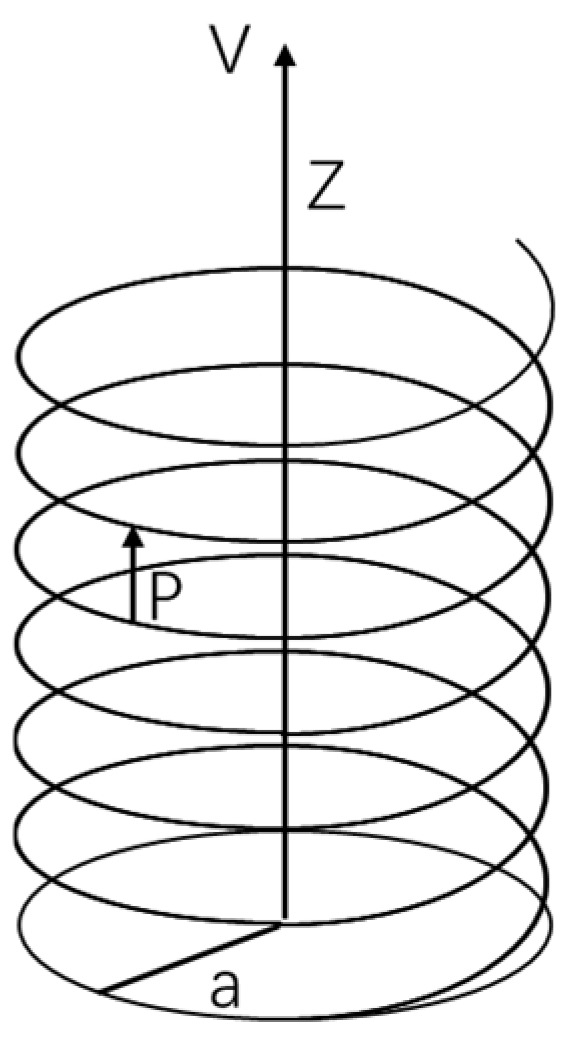
Helical motion of electrons under the potential field of chiral molecules.

As shown in Figure 2, an electron moves in a helical trajectory along the Z-axis with a velocity V under the potential field of chiral molecules. The helical motion has a radius of a and a pitch of p. The equation of motion for the electron in this coordinate system can be written as:

$$\left\{\begin{array}{c} x=a\cdot \cos\left(\frac{2\pi z}{p}\right)\\ y=a\cdot \sin\left(\frac{2\pi z}{p}\right)\\ z=v\cdot t \end{array}\right.$$

The second derivative is as follows:

$$\left\{\begin{array}{c} \ddot{x}=-a{\left(\frac{2\pi v}{p}\right)}^{2}\cos\left(\frac{2\pi z}{p}\right)\\ \ddot{y}=-a{\left(\frac{2\pi v}{p}\right)}^{2}\sin\left(\frac{2\pi z}{p}\right)\\ \ddot{z}=0 \end{array}\right.$$

Based on the above equation and the classical force and effective spatial potential formula:

$$\vec{F}=m\vec{\ddot{r}}=-\nabla V$$

We can introduce them into the Hamiltonian operator of linear momentum and spin coupling:

$$H_{SO}=-\frac{\hbar }{4mv^{2}}\vec{\sigma }\cdot \vec{\dot{r}}\times \nabla V$$

Thus, the expression for *H_SO_* can be derived. Using the commutation relations:

$$\frac{d\vec{\sigma }\left(t\right)}{dt}=\frac{i}{\hbar }\left[H_{SO},\vec{\sigma }\left(t\right)\right]$$

We can determine the changes in spin during the transport process under spin–orbit coupling, as given by the following equations:

(1) $$\left\{\begin{array}{c} \frac{d\sigma_{x}}{d\theta }=-b\sigma_{z}\cos\theta -2g^{2}\sigma_{y}\\ \frac{d\sigma_{y}}{d\theta }=-b\sigma_{z}\sin\theta +2g^{2}\sigma_{x}\\ \frac{d\sigma_{z}}{d\theta }=b\left(\sigma_{y}\sin\theta +\sigma_{x}\cos\theta \right) \end{array}\right.$$

Here, *θ* represents the angle with respect to the Z-axis. *g* and *b* are coefficients related to the pitch *p*, radius *a*, and electron velocity *V*:

(2) $$\left\{\begin{array}{c} b=\frac{\pi av^{2}}{pc^{2}}\\ g=\frac{\pi av}{cp} \end{array}\right.$$

It seems that Equations (1) and (2) are able to explain the CISS effect well enough despite being derived with a semi-classical model. Equation (1) shows that the electron’s spin changes with the variation of the *θ* angle, implying that the spin distribution along the helical path is not uniform. Equation (2) demonstrates that the sign of the pitch *p* changes upon molecular chirality inversion, indicating the correlation between the CISS effect and molecular chirality. Through these two equations, it can be observed that electrons with spin pointing upward appear to preferentially pass through left-handed chiral molecules while electrons with spin pointing downward tend to pass through right-handed chiral molecules. This observation is consistent with the experimental results.

**Figure 3:**
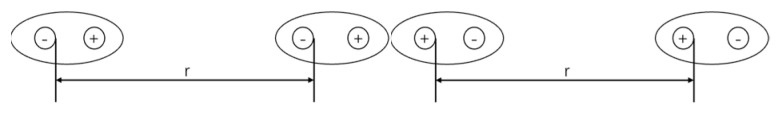
Schematic of the Van der Waals–London forces.

As shown in Figure 3, when two organic molecules approach each other, an electric dipole moment is induced, causing the charge centers of the reacting molecules to separate. The total energy of this system comprises the kinetic energy of the two molecules and the Coulomb potential energy. Therefore, the zero-point vibrational energy of the system corresponds to the Coulomb potential energy of the two molecules. This zero-point vibrational energy can be approximated as:

(3) $$E{’}_{0}=h\gamma_{0}-\frac{\alpha^{2}}{32\pi^{2}r^{6}}h\gamma_{0}$$

where the molecular polarizability coefficient *α* is defined as:

(4) $$\alpha =\frac{q^{2}}{\varepsilon_{0}k}$$

From the expression of *E*’_0_, it can be observed that the Van der Waals–London forces are attractive in nature. Furthermore, this attractive force rapidly increases as the molecules approach each other and is generated by the movement of charges within the two molecules. As a result, as the molecules come closer, the charge centers within the molecules deviate further from their original positions. This indicates that for point-neutral molecules, the internal charge distribution undergoes reorganization before a chemical reaction occurs. It is only when bonding begins that the deviation of charge centers within the molecules reaches its maximum.

### 3.2. Chiral Selectivity of the AADC Enzyme

As is widely known, the AADC enzyme plays a crucial role in neural regulation in the human body. The AADC enzyme belongs to a class of enzymes known as chiral enzymes, which exhibit high enantioselectivity toward chiral substrates (such as L-amino acids), meaning they catalyze reactions selectively with one enantiomer over the other.

**Figure 4:**
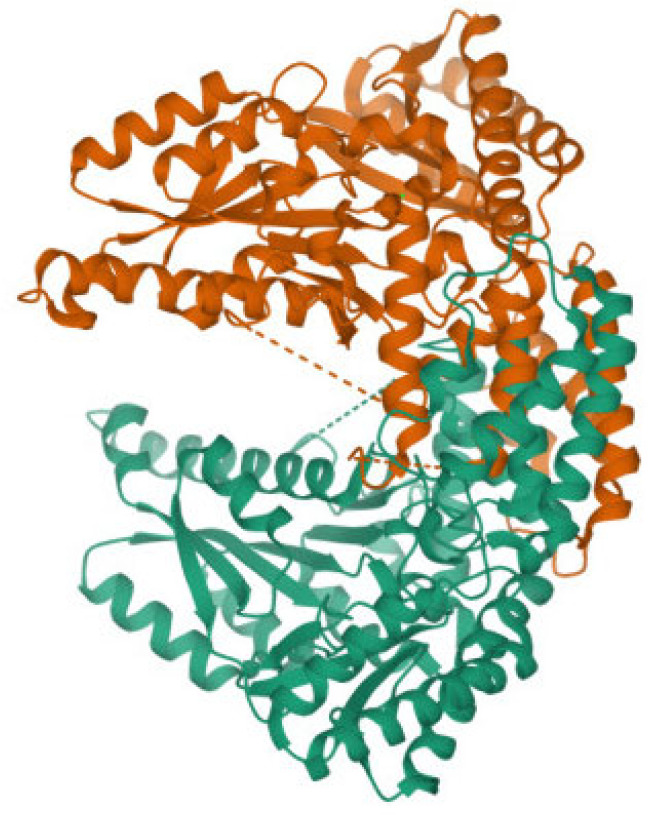
Ribbon diagram of the AADC enzyme.

As shown in Figure 4, the AADC enzyme consists of two peptide chains, the A-chain and B-chain, arranged symmetrically in a chiral manner. However, all the helical structures forming these peptide chains are levorotatory, suggesting that the amino acid molecules constituting these peptide chains are also entirely levorotatory. Therefore, AADC enzyme is a type of levorotatory enzyme.

**Figure 5:**
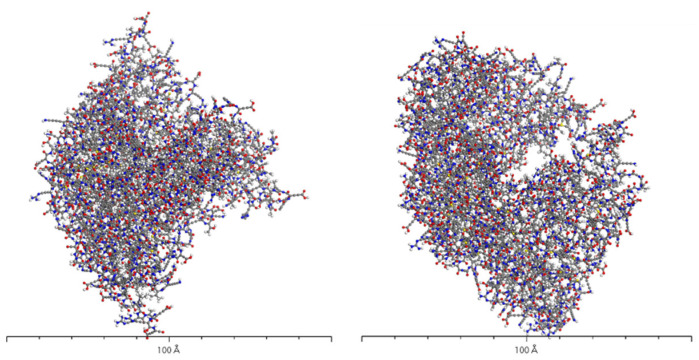
Ball-and-stick model of the AADC enzyme.

Figure 5 illustrates the structure of the AADC enzyme after structural optimization using the Forcite algorithm. From the figure, it is evident that the structure of AADC enzyme in Material Studio is extremely intricate, making it difficult to visually observe the folding and bending processes of the protein. However, in an overall perspective, the shapes of Figure 4 and Figure 5 are consistent. At the edges of AADC enzyme, there are some long carbon chains present on its surface.

**Figure 6:**
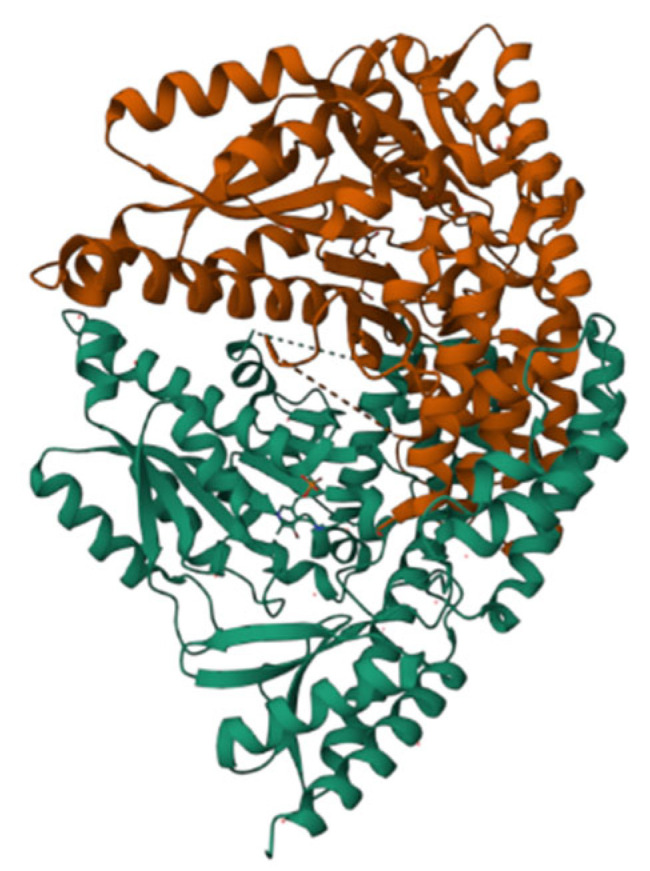
Ribbon diagram of AADC enzyme complexed with PLP and LLP.

**Figure 7:**
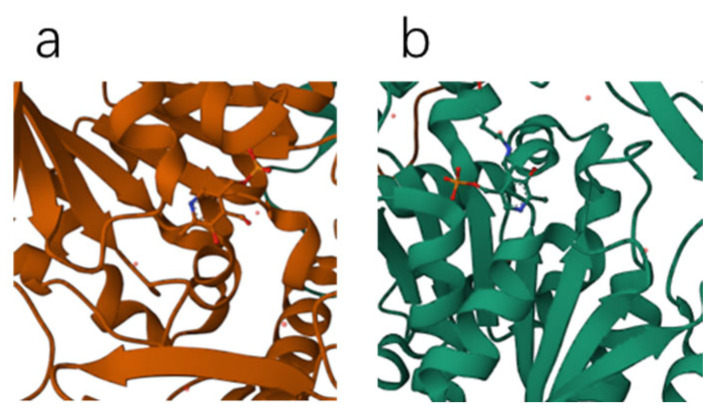
PLP ligand (**a**) and LLP ligand (**b**).

Figure 6 illustrates the structures of LLP and PLP ligands formed on the AADC enzyme. As evident from Figure 7, both LLP and PLP ligands possess a benzene ring structure. These ligands are situated on opposite sides of the central crevice of the AADC enzyme. Consequently, the catalytic site for the conversion of L-DOPA is located at the vicinity of these ligands. The region surrounding these ligands is encompassed by protein structures with levorotatory conformations, creating a relatively enclosed reaction environment that favors the preservation of proper product molecular structures.

### 3.3. L-DOPA and D-DOPA

**Figure 8:**
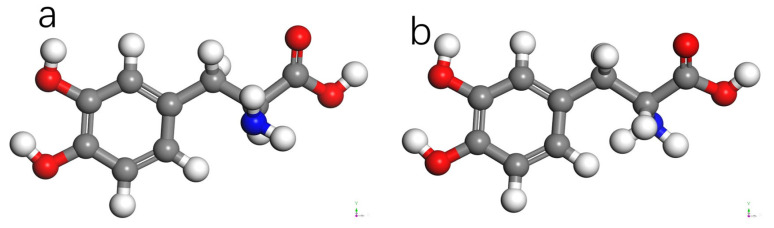
Molecular structure of DOPA. (**a**) L-DOPA. (**b**) D-DOPA.

Figure 8 represents the optimized structures of the two chiral forms of dopamine molecules using the DMol3 algorithm [17,18]. The chirality of these molecules arises from the presence of three different substituents attached to the third carbon atom of the benzene ring, leading to the optical rotation and other factors that contribute to the chiral selectivity of AADC enzyme.

### 3.4. Molecular Dynamics Simulation of Dopamine Molecules with AADC Enzyme

In order to simulate the real interaction between AADC enzyme and dopamine molecules in the human body, we employed the Monte Carlo algorithm to randomly place dopamine molecules in the vicinity of the AADC enzyme and searched for the configurations that minimized the overall system energy. We also set up several control groups with different quantities of L- (or D-) dopamine molecules, including 32, 100, 200, and 300, interacting with one AADC enzyme. The system temperature during the calculations was set to 310 K (approximately the normal body temperature), and 30 frames were output at each calculation. According to the study by Rahman, M. K. and Nagatsu [19], when the concentration of L-DOPA is 414 μM, the reaction rate of AADC enzyme reaches half of its maximum velocity. Therefore, we conservatively established systems with 32, 100, and 200 molecules of L-DOPA and the AADC enzyme for simulation calculations.

**Figure 9:**
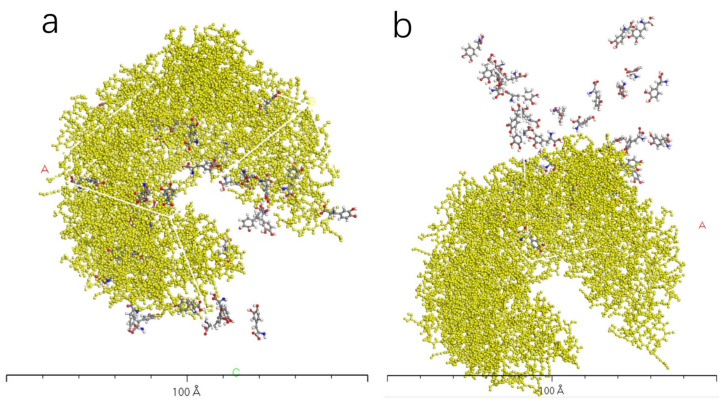
The AADC enzyme with 32 L-DOPA (**a**) and 32 D-DOPA (**b**).

**Figure 10:**
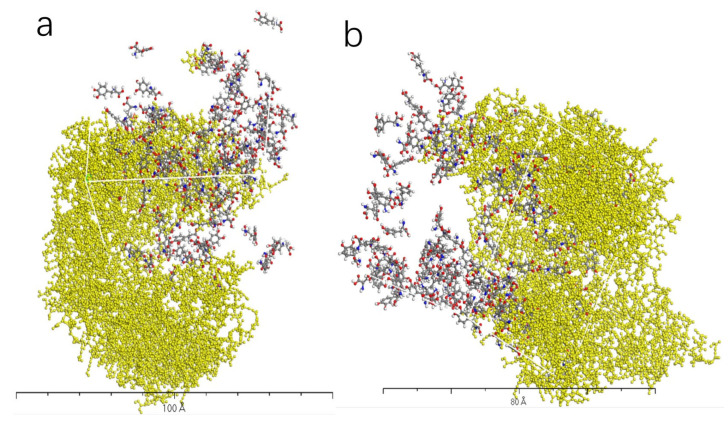
The AADC enzyme with 100 L-DOPA (**a**) and 100 D-DOPA (**b**).

**Figure 11:**
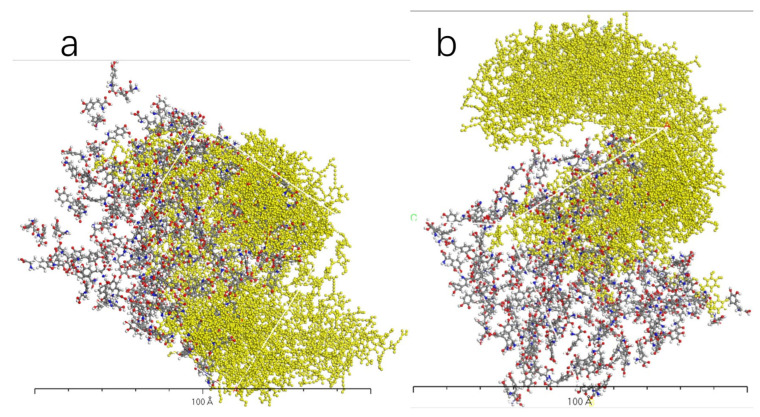
The AADC enzyme with 200 L-DOPA (**a**) and 200 D-DOPA (**b**).

**Figure 12:**
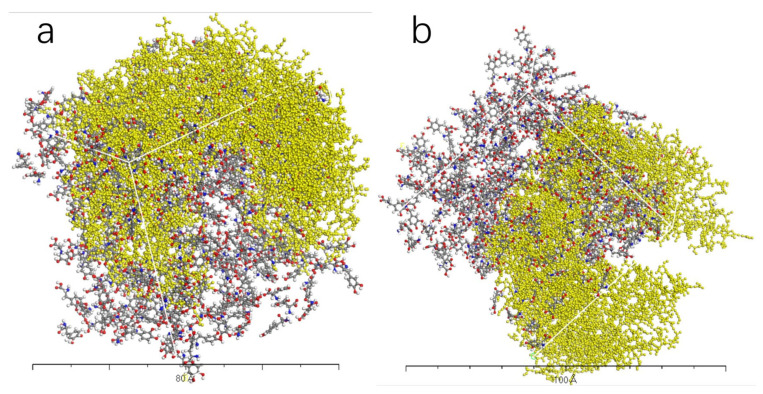
The AADC enzyme with 300 L-DOPA (**a**) and 300 D-DOPA (**b**).

Figure 9, Figure 10, Figure 11 and Figure 12 represent the outcomes of the dynamic simulation. The left-hand images depict the interaction between L-DOPA and the AADC enzyme, while the right-hand images illustrate the interaction between D-DOPA and the AADC enzyme. The AADC enzyme molecules are marked in yellow. As analyzed in Section 3.2, the active site for the interaction between the AADC enzyme and DOPA molecules is located at the central concave region of the enzyme. From these four sets of images, it can be observed that D-DOPA exhibited a repulsive behavior relative to L-DOPA when interacting with the AADC enzyme molecules. Most D-DOPA molecules tended to distribute themselves on the opposite side of the AADC enzyme, away from the active site, whereas L-DOPA molecules tended to cluster around the concave region of the AADC enzyme. This indicates that D-DOPA molecules seem to be less inclined to react with the AADC enzyme.

For more information, we calculated the total energy for each system.

**Figure 13:**
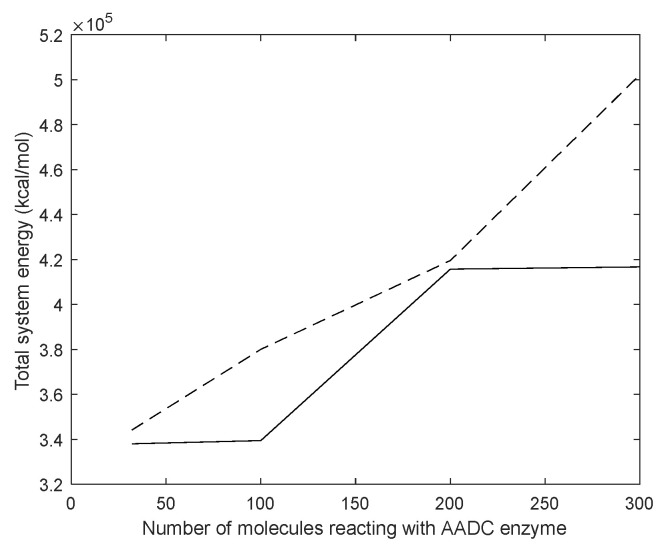
Total energy of the system consisting of D-DOPA (- -) and AADC in comparison to that comprising L-DOPA (—) and the AADC enzyme.

From Figure 13, it can be observed that the total energy of the system consisting of D-DOPA and the AADC enzyme was consistently higher than that of the system comprising L-DOPA and the AADC enzyme. This indicates that the interaction energy between L-DOPA and the AADC enzyme is smaller compared to the interaction between D-DOPA and the AADC enzyme.

### 3.5. Charge Transfer in the Interaction of L-DOPA and D-DOPA with Peptide Chains

To validate the hypothesis mentioned above, it is necessary to calculate the charge transfer between the enzyme molecule and L-DOPA, as well as D-DOPA, during the occurrence of the reaction. For the convenience of computation, we calculated the charge transfer between levorotatory peptide chain molecules consisting of different numbers of amino acid units and L-DOPA or D-DOPA molecules. Furthermore, we calculated the Fukui indices of L-DOPA and D-DOPA molecules using the DMol3 [17,18] module in MS to determine the active sites. The Fukui index provides a qualitative judgment of which functional group in the molecule is most likely to be involved in a chemical reaction. The larger the Fukui index of an atom, the higher its chemical reactivity will be. The Fukui index is categorized into nucleophilic attack (f+), electrophilic attack (f−), and radical attack (f0). The calculated results of the electrophilic attack for L-DOPA and D-DOPA molecules are shown below.

**Figure 14:**
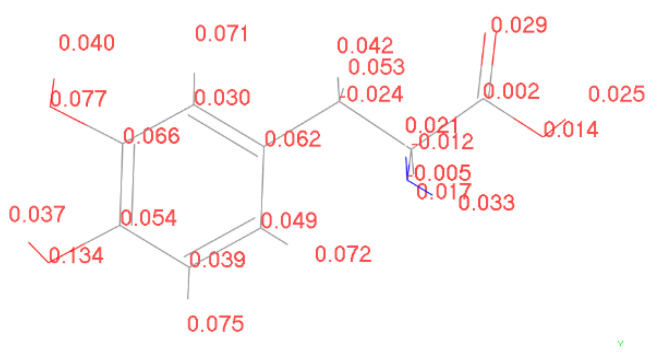
Electrophilic attack of L-DOPA.

**Figure 15:**
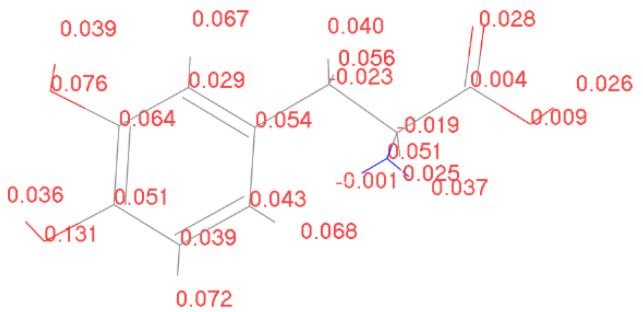
Electrophilic attack of D-DOPA.

Figure 14 and Figure 15 reveal shared susceptible electron loss sites in L-DOPA and D-DOPA, situated at the oxygen atom of the benzene ring’s lower-left portion. The electrophilic attack stands at 0.134 and 0.131 for L-DOPA and D-DOPA, respectively.

Guided by these findings, we positioned the benzene ring of both compounds proximal to the side of the levorotatory peptide chain in subsequent calculations. This alignment ensured their adjacency. Furthermore, levorotatory peptide chains of varying lengths (10, 15, 20, and 25 amino acid units) were configured, maintaining consistent pitch and radius for each chain. The molecular approach during the chemical reaction was simulated by determining the centers’ distances between L-DOPA/D-DOPA molecules and the peptide chain molecules. Given distinct functional groups at the peptide chain ends, discrete considerations were required. Therefore, separate assessments were conducted for the N-terminus and C-terminus scenarios as they interacedt with L-DOPA and D-DOPA molecules.

**Figure 16:**
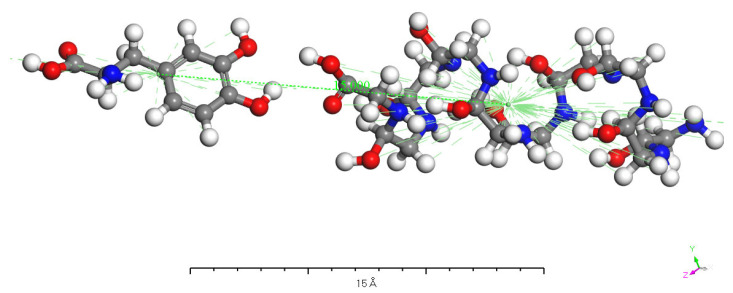
D-DOPA approaching the C-terminus of a peptide chain composed of 10 amino acids.

Figure 16 illustrates a computed charge transfer model from earlier discussions, depicting the scenario where the benzene ring side of the D-DOPA molecule approaches the C-terminus of a 10-amino acid levorotatory peptide chain. The initial distance between the molecule centers was set at 15 angstroms. This distance, however, is preliminary, subject to structural optimization before electron population calculations. Structural optimization leads to the lowest energy state for both molecules, reflecting the most probable real-world configuration. The intermolecular distance will be adjusted accordingly.

Due to the plethora of atoms and their valence electrons, it is difficult to discern the trend of charge transfer by directly analyzing the charge transfer data of each atom. Therefore, a method akin to calculating the center of mass of an object can be used to compute the positions of the positive and negative charge centers of the molecules. The calculation formula is as follows:

$$\bar{{\vec{r}}_{+}}=\frac{\sum q_{+}\left(i\right){\vec{r}}_{+}\left(i\right)}{\sum q_{+}\left(i\right)}$$

$$\bar{{\vec{r}}_{-}}=\frac{\sum q_{-}\left(i\right){\vec{r}}_{-}\left(i\right)}{\sum q_{-}\left(i\right)}$$

After performing the above calculation for each molecule, the positions of the positive and negative charge centers of the molecules were obtained. Then, the difference between the two position vectors was calculated and the magnitude of the resulting vector represents the distance between the positive and negative centers of the molecule in that specific scenario, which indicates the polarization strength of the molecule.

**Figure 17:**
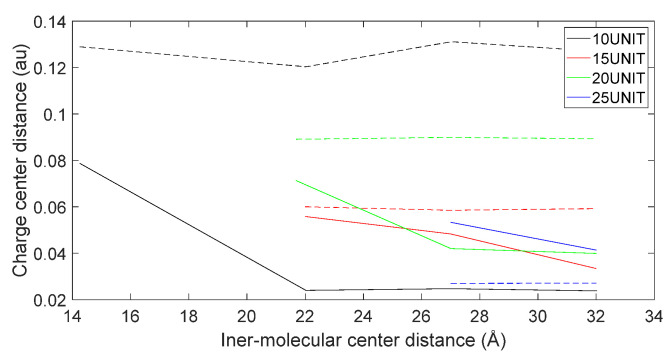
Variation in distance as D-DOPA (—) approaches the C-terminus of the peptide chain (- -).

**Figure 18:**
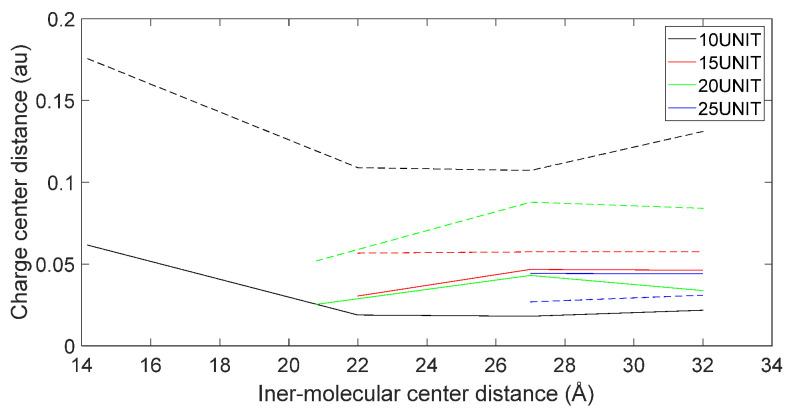
Variation in distance as D-DOPA (—) approaches the N-terminus of the peptide chain (- -).

**Figure 19:**
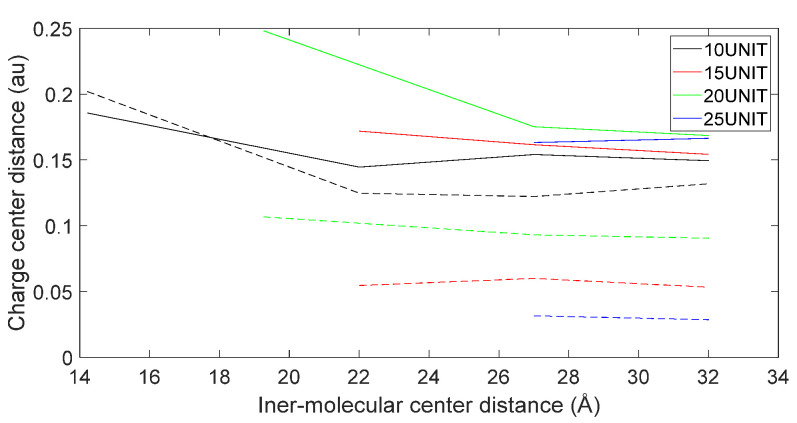
Variation in distance as L-DOPA (—) approaches the C-terminus of the peptide chain (- -).

**Figure 20:**
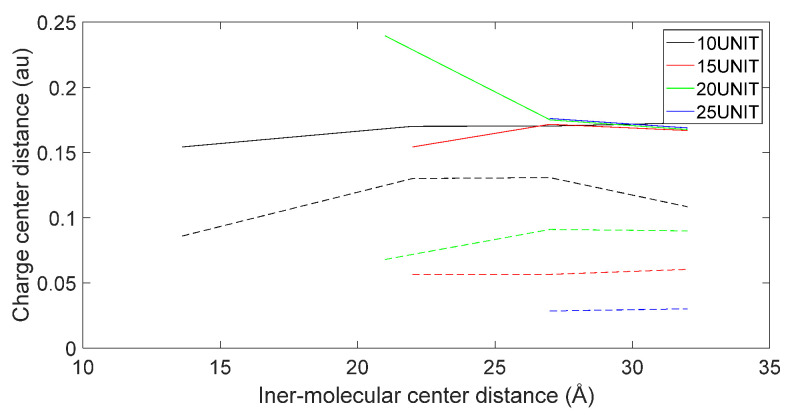
Variation in distance as L-DOPA (—) approaches the N-terminus of the peptide chain (- -).

Figure 17, Figure 18, Figure 19 and Figure 20 reveal notable distinctions in the distances between positive and negative charge centers. Specifically, D-DOPA demonstrated a smaller inter-center distance than the peptide molecule, while L-DOPA exhibited a larger distance. Moreover, the displacement of positive and negative charge centers was more pronounced in L-DOPA than D-DOPA. These findings underscore L-DOPA’s higher susceptibility to polarization, with marginal variation between the peptide molecule charge centers in the L-DOPA and D-DOPA comparison groups. This divergence is attributed to the peptide molecule’s substantial molecular weight relative to L-DOPA and D-DOPA, resulting in a greater electron count. Consequently, distance alterations between the molecules exerted a more pronounced impact on the peptide molecule’s charge center variation than the effects introduced by differing DOPA molecules.

By calculating the charge distribution of the molecules in their individual states and when in proximity to each other, and subsequently taking the difference between the two results, we can obtain the absolute value of charge transfer during the approaching process of the molecules. The computational results are presented as follows.

**Figure 21:**
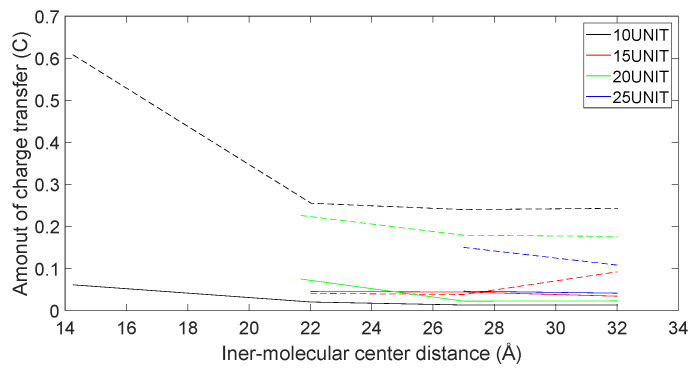
The charge transfer as D-DOPA (—) approaches the C-terminus of the peptide chain (- -).

**Figure 22:**
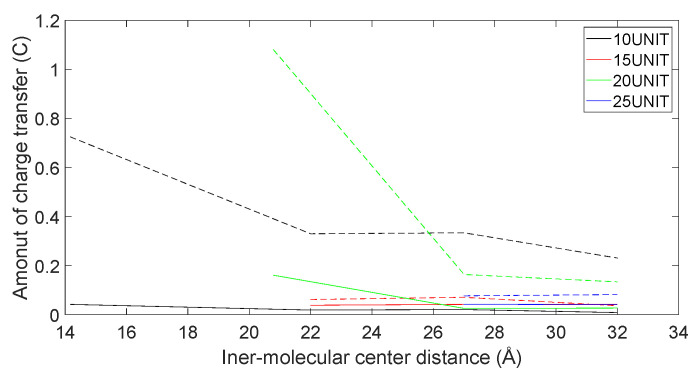
The charge transfer as D-DOPA (—) approaches the N-terminus of the peptide chain (- -).

**Figure 23:**
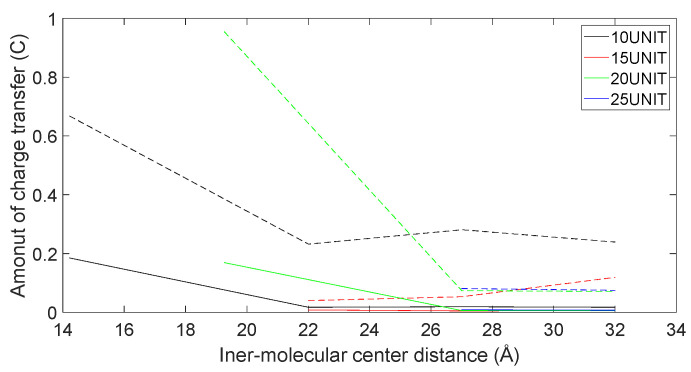
The charge transfer as L-DOPA (—) approaches the C-terminus of the peptide chain (- -).

**Figure 24:**
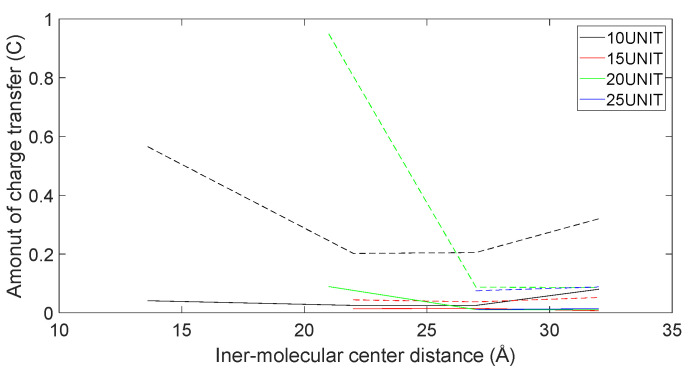
The charge transfer as L-DOPA (—) approaches the N-terminus of the peptide chain (- -).

In Figure 21, Figure 22, Figure 23 and Figure 24, the following conclusions emerge: First, D-DOPA molecules exhibited an increased charge transfer with a higher peptide chain molecular weight, while L-DOPA molecules displayed a reduced charge transfer with a lower peptide chain molecular weight. Second, the charge transfer in the peptide chain, whether with L-DOPA or D-DOPA, rose as the inter-molecular distance decreased. Additionally, the peptide chain’s charge transfer generally surpassed that of individual L-DOPA or D-DOPA molecules. Furthermore, D-DOPA molecules exhibited greater charge transfer propensity compared to L-DOPA, in line with the charge center analysis. Decreasing inter-molecular distance accentuated the charge transfer distinctions between the peptide chain and L-DOPA, D-DOPA molecules.

These findings affirm that molecules with more charges manifest increased internal charge transfers during chemical reactions. The substantial molecular weight of the enzyme molecule underscores a significant manifestation of the CISS effect during chemical reactions.

### 3.6. Reaction Trends and CISS Effect

Previously, we examined the charge transfer trends of L-DOPA and D-DOPA toward peptide chains of varying lengths. For finer insights, we performed separate calculations utilizing electronic charge distribution data to model interactions between a 10-amino acid peptide and both L-DOPA and D-DOPA. The outcomes yielded spatial representations of the positions of positive and negative charge centers as the molecules approached each other.

**Figure 25:**
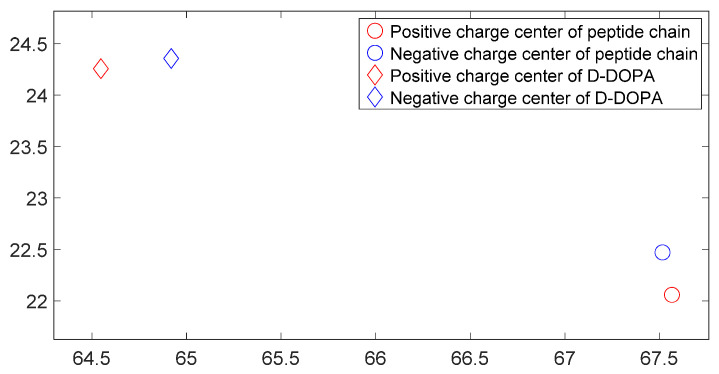
The distribution of charge centers as D-DOPA approaches the C-terminus of the peptide chain.

**Figure 26:**
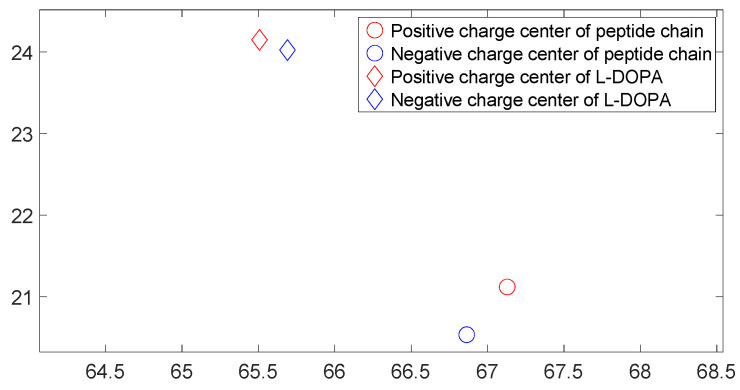
The distribution of charge centers as L-DOPA approaches the C-terminus of the peptide chain.

**Figure 27:**
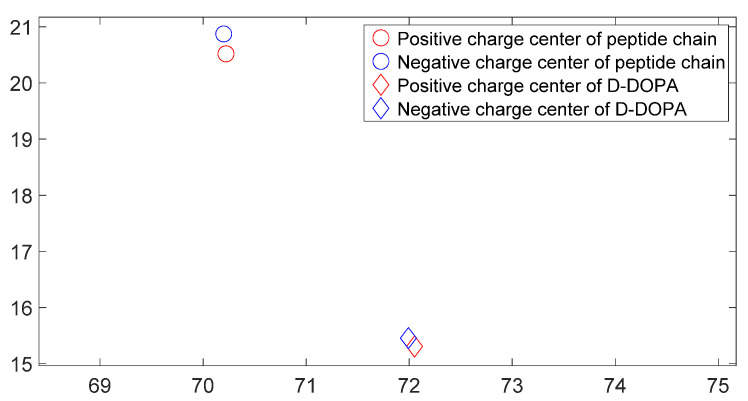
The distribution of charge centers as D-DOPA approaches the N-terminus of the peptide chain.

**Figure 28:**
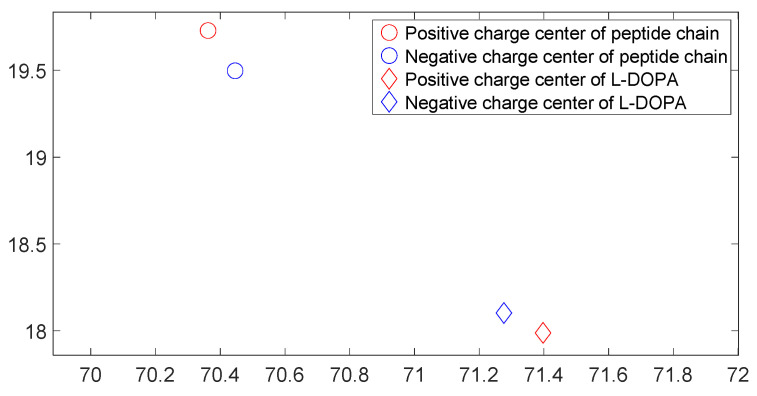
The distribution of charge centers as L-DOPA approaches the N-terminus of the peptide chain.

Figure 25, Figure 26, Figure 27 and Figure 28 illustrate charge center positions with consistent intermolecular distances, projected on the X-Y plane within three-dimensional space. Notably, Figure 25 and Figure 26 highlight a significant angle between lines linking interacting charge centers. This suggests the C-terminus of the polypeptide chain exerts repulsion on both L-DOPA and D-DOPA, favoring their reaction with the polypeptide’s N-terminus. Both L-DOPA and D-DOPA exhibited negative charge center approaches to the polypeptide, aligning with predictions based on Fukui index. D-DOPA notably targeted the positive charge center of the polypeptide, while L-DOPA approached its negative charge center. This conforms to the Section 3.5 analysis, signifying L-DOPA’s propensity to form covalent bonds via electron sharing and D-DOPA’s approach to the polypeptide’s positive charge center due to charge-spin repulsion. The latter case results in parallel spin electrons transferring to the molecule’s negative charge center upon polypeptide interaction, while compatible spin electrons remain at the positive charge center.

**Figure 29:**
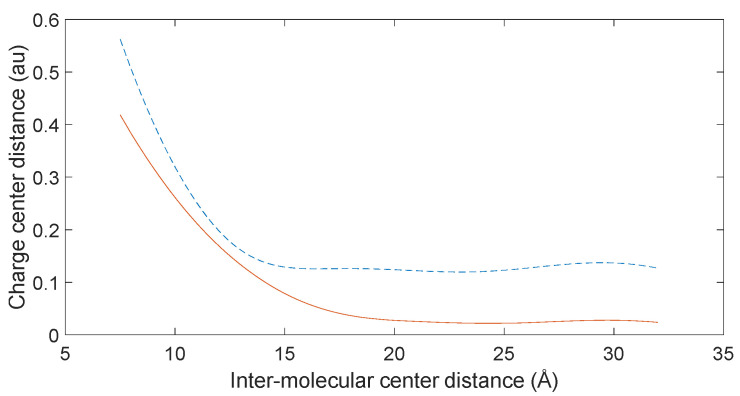
Variation of the charge center distance between the D-DOPA molecule (—) and peptide chain (- -).

Utilizing electronic population calculation data for the interaction between the C-terminus of a 10-amino acid polypeptide molecule and D-DOPA, we employed polynomial fitting via the least-squares method to establish the relationship between the distance of their positive and negative charge centers and the overall intermolecular distance. Figure 29 illustrates that as the molecules were initially distant, their charge center distance remained minimal; however, this distance abruptly increased upon reaching a certain proximity.

Equations (3) and (4) in Section 3.1 highlight that attractive intermolecular forces between nonpolar molecules intensify with molecular polarization, accelerating their approach during reactions. As Figure 29 suggests, rapid molecule approach corresponds to a swift increase in charge center distance. Since stationary positively charged atoms in nonpolar molecules are involved, electron transfer speed escalates with their proximity.

In cases where one molecule possesses a chiral structure, Equations (1) and (2) in Section 3.1. reveal that heightened electron transfer speed leads to a notable CISS effect.

Consequently, with chiral molecules as reactants, the CISS effect prevails prominently when close, while its manifestation wanes when distant.

## 4. Discussion

### 4.1. Dynamic Simulation Analysis

Section 3.4 indicates greater interaction energy between the AADC enzyme and D-DOPA molecules compared to the L-DOPA molecules, hinting at the AADC enzyme’s chiral selectivity. L-DOPA and D-DOPA share identical chemical structures, implying equivalent valence electron arrangements and chemical properties. This distinction is primarily attributed to the electron spin of L-DOPA and D-DOPA molecules.

According to the theoretical analysis presented in Section 3.1, non-polar molecules undergo polarization currents during chemical reactions, resulting in electron redistribution within the molecules, whereas the AADC enzyme is a levorotatory protein molecule. Based on the CISS effect, valence electrons with spin-up are more likely to accumulate near the negative charge center of the molecule, while valence electrons with spin-down are more distributed around the positive charge center. For stable, low-energy chemical bonding with the AADC enzyme, the spin direction of the reactant’s valence electrons must be antiparallel to that of the AADC enzyme. Since covalent bond formation involves the sharing of electron pairs, molecules that can form stable structures with the AADC enzyme must carry valence electrons with the opposite spin direction to the negative charge center of AADC, i.e., spin-down valence electrons. Conversely, substances that form unstable interactions with the AADC enzyme are expected to carry a higher proportion of spin-up valence electrons.

Therefore, it is conjectured that L-DOPA carries spin-down valence electrons, fostering covalent bonds with AADC and anchoring at its catalytic site. Conversely, D-DOPA’s spin-up valence electrons require a transition to a triplet state surface for AADC interactions, forming an unstable bond through electron exchange. This process increases interaction potential energy and yields an energy barrier. This barrier signifies energy needed for valence electron transition to the nearest orbital. Such spin differences lead to AADC’s chiral selectivity. Moreover, when the enzyme spin has a preferred direction, reduced charge recombination improves the charge transfer and enhances reaction rates [20].

### 4.2. Spin Theoretical Analysis and Verification of L-DOPA and D-DOPA

Based on the theoretical analysis in the preceding section, L-DOPA is likely to carry more downward-oriented spin electrons, while D-DOPA may carry more upward-oriented spin electrons. To validate this, we utilized the CASTEP [21] computational package, factoring in spin–orbit interactions for enhanced accuracy. However, it should be noted that CASTEP exclusively supports calculations for crystal cell structures.

**Figure 30:**
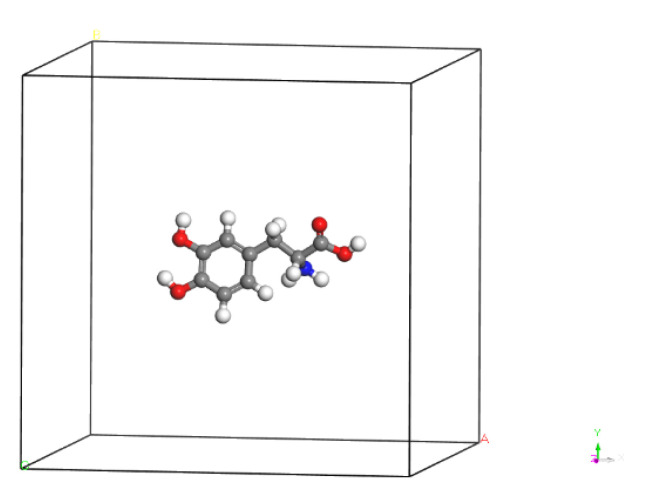
A crystal cell containing one D-DOPA molecule.

As illustrated in Figure 30, we placed L-DOPA and D-DOPA molecules in sufficiently large crystal cells, ensuring that the intermolecular interactions could be neglected during the computations.

**Figure 31:**
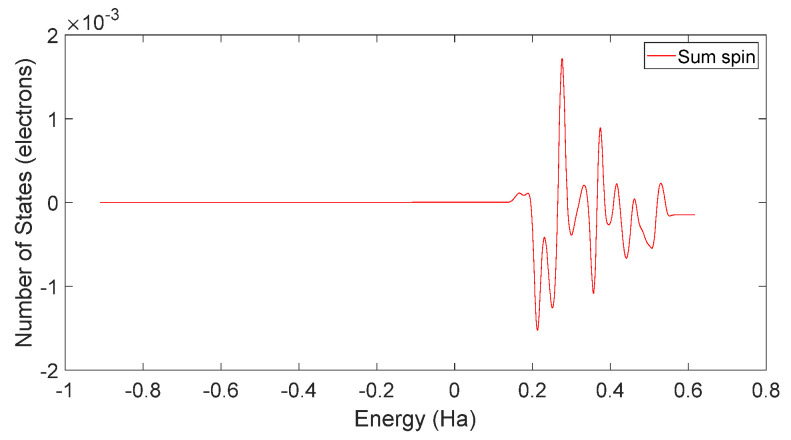
Total spin-state density in L-DOPA.

**Figure 32:**
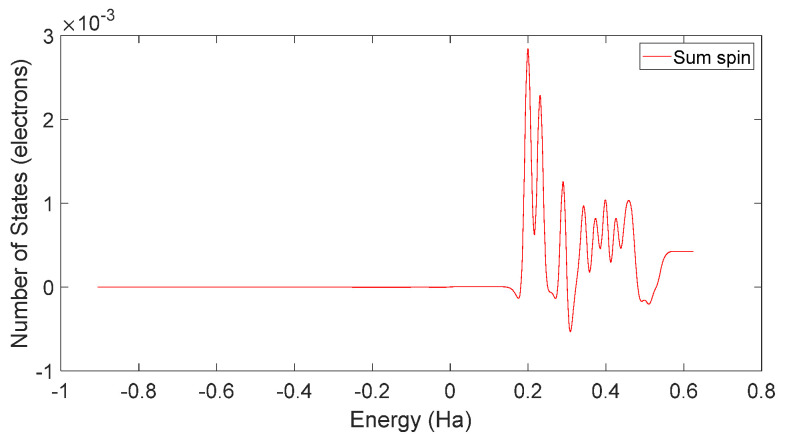
Total spin-state density in D-DOPA.

Based on Figure 31 and Figure 32, it can be observed that in L-DOPA molecules, the majority of higher-energy electrons had a spin orientation predominantly downward, while in D-DOPA molecules, the majority of higher-energy electrons had a spin orientation predominantly upward. This indicates that in L-DOPA molecules, the majority of valence electrons have a spin orientation downward, whereas in D-DOPA molecules, the majority of valence electrons have a spin orientation upward.

**Figure 33:**
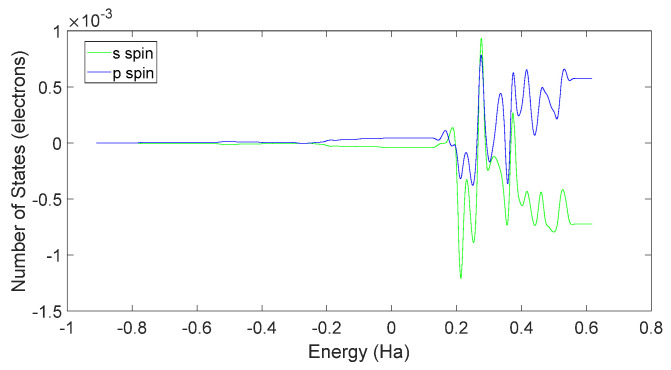
Spin-state density of S orbital and P orbital in L-DOPA.

**Figure 34:**
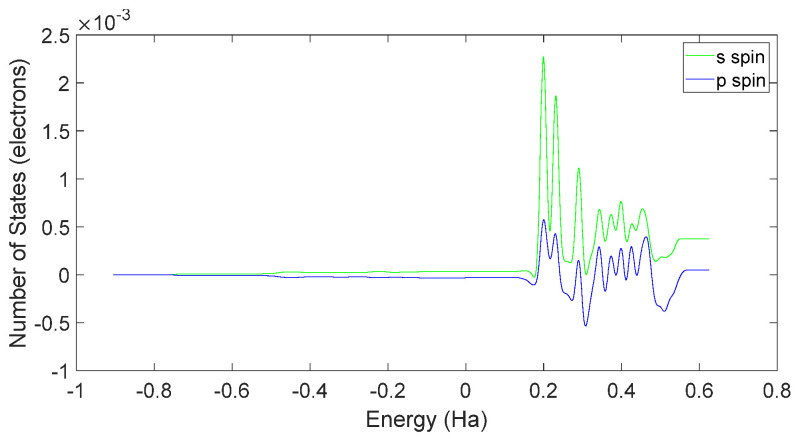
Spin-state density of S orbital and P orbital in D-DOPA.

**Figure 35:**
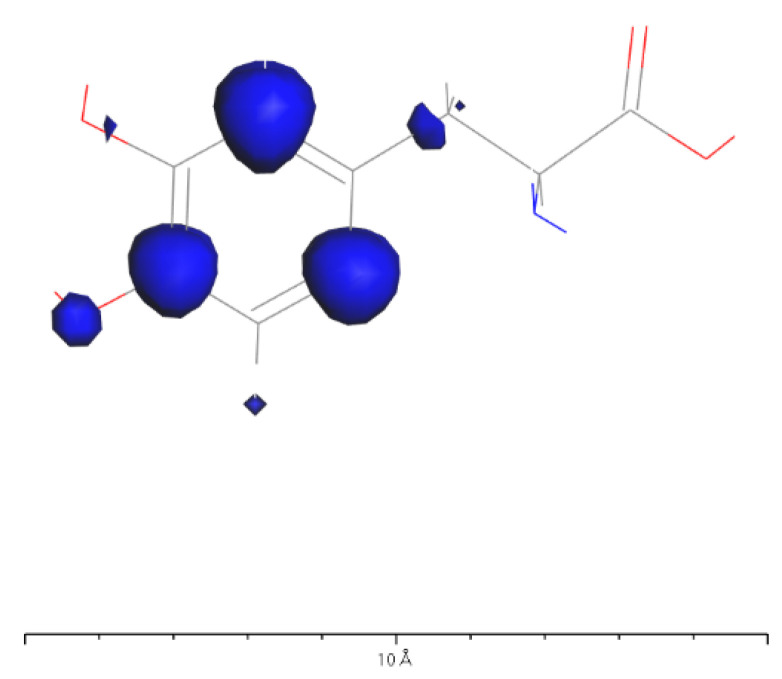
Spin electron distribution in the L-DOPA molecule.

**Figure 36:**
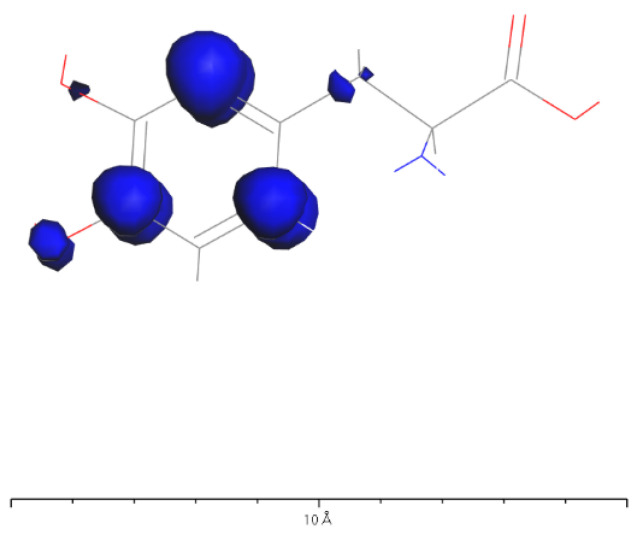
Spin electron distribution in the D-DOPA molecule.

Further examination of Figure 33 and Figure 34 reveals that the downward spin orientation of valence electrons in L-DOPA molecules was mainly contributed by the P orbital, whereas the upward spin orientation of valence electrons in D-DOPA molecules was jointly provided by the S and P orbitals. As demonstrated in Figure 35 and Figure 36, the atoms primarily responsible for providing the spin-polarized electrons were oxygen and carbon atoms. Among these, the oxygen atom identified in Section 3.5 using Fukui indices analysis was included. The difference in electron spin orientations between L-DOPA and D-DOPA molecules is also consistent with the optical rotation of chiral substances.

In chemistry, chiral molecules often display optical rotation, a phenomenon well studied in the field. Numerous theories have elucidated optical rotation [22]. When subjecting a solution of L-DOPA to polarized light, the light’s polarization plane rotates leftward, whereas with a solution of D-DOPA, the rotation is rightward.

Importantly, this optical rotation does not necessitate an external magnetic field, distinguishing it from energy level splitting under such conditions. A polarized light beam combines left-handed and right-handed circularly polarized light. When their frequencies and intensities match, the resulting beam’s polarization plane remains unaffected. Optical rotation in L-DOPA and D-DOPA results from their distinct abilities to absorb left-handed and right-handed circularly polarized light.

**Figure 37:**
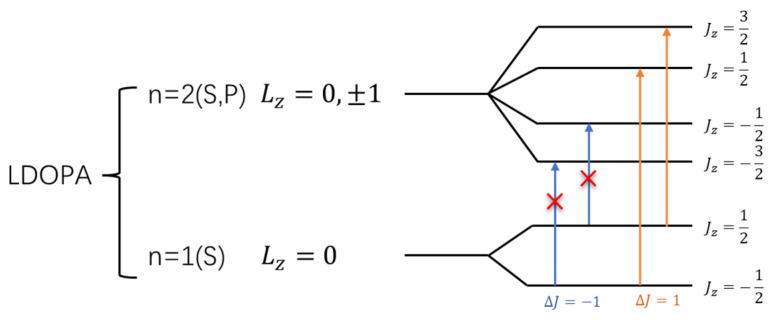
Schematic of the electron angular momentum in L-DOPA.

**Figure 38:**
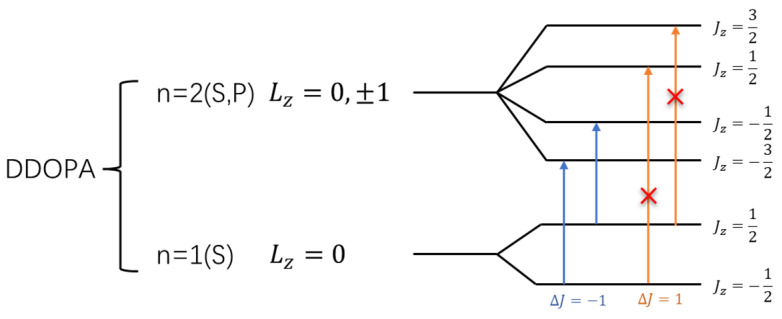
Schematic of the electron angular momentum in D-DOPA.

**Figure 39:**
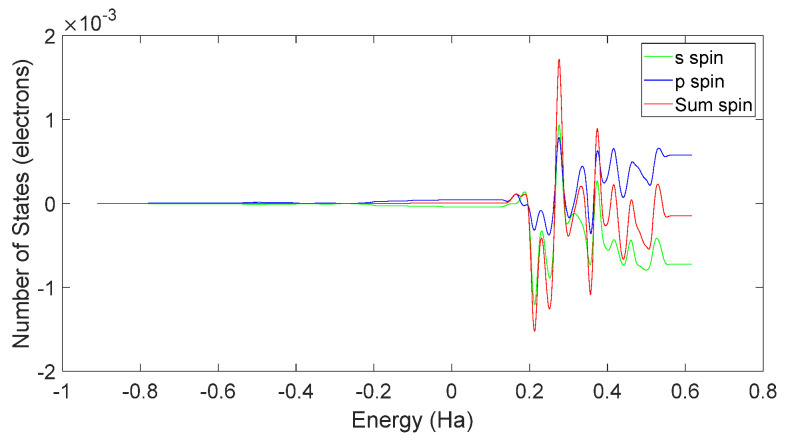
Electron spin-state density in L-DOPA.

**Figure 40:**
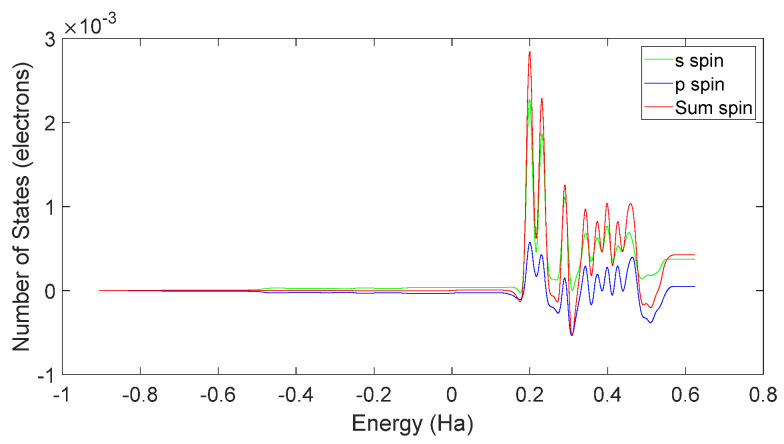
Electron spin-state density in D-DOPA.

In both L-DOPA and D-DOPA molecules, the prominent atoms are oxygen and carbon with relatively high atomic numbers. Their electronic configurations are as follows: Oxygen—1*S*^2^2*S*^2^2*P*^4^, Carbon—1*S*^2^2*S*^2^2*P*^2^. The corresponding orbital energy levels are shown in Figure 37 and Figure 38, where electrons carried both orbital angular momentum *L_Z_* and spin $\sigma =\pm \frac{1}{2}$, forming the total angular momentum *J_Z_*, leading to the energy level diagram. The depicted coupled energy levels in the figure pertain to the molecule, considering the absence of an external magnetic field to induce the Zeeman effect. Therefore, the energy levels in Figure 37 and Figure 38 are not separated by energy differences. Figure 39 and Figure 40 serve as a summary of Figure 31, Figure 32, Figure 33 and Figure 34. Through Figure 39 and Figure 40, one can more clearly discern the relationship between the electron spin distribution of each orbital and the overall electron spin distribution across the two molecules.

Light absorption occurs when incoming light matches energy level differences. Although electronic transitions can occur within the same principal quantum number, these transitions do not absorb light due to their minimal energy disparity. Consequently, light absorption transitions arise between energy levels with distinct principal quantum numbers, specifically from the n = 1 to n = 2 levels, as illustrated in the figure.

However, when electrons interact with photons, they must satisfy the conservation of angular momentum. Therefore, after absorbing right-circularly polarized light, electrons will undergo transitions with Δ*J* = 1, as indicated by the orange arrows in Figure 37 and Figure 38. Similarly, after absorbing left-circularly polarized light, electrons will undergo transitions with Δ*J* = −1, as indicated by the blue arrows in Figure 37 and Figure 38.

When the atomic energy level possesses a total electron spin of 0, it equally absorbs left-circularly polarized and right-circularly polarized light. As demonstrated in Figure 31, Figure 32, Figure 33 and Figure 34, Figure 37 reveals that in the L-DOPA molecule, the majority of spin-down valence electrons occupied states with $J_{z}=-\frac{1}{2}$ and $J_{z}=-\frac{3}{2}$. Similarly, in Figure 38 for D-DOPA, most of the spin-up valence electrons occupied states with $J_{z}=\frac{1}{2}$ and $J_{z}=\frac{3}{2}$. Due to the Pauli exclusion principle, these occupied states cannot accept additional electrons, thus inhibiting these transitions. As a result, the transitions marked with red crosses on the arrows in Figure 37 and Figure 38 are less likely for these molecules.

From the figures, it can be observed that L-DOPA molecules are more inclined to undergo transitions with Δ*J* = 1, which means they are more likely to absorb right-circularly polarized light. On the other hand, D-DOPA molecules are more prone to undergo transitions with Δ*J* = −1, making them more likely to absorb left-circularly polarized light.

Preferential absorption of right-circularly polarized light by L-DOPA reduces the intensity of such light upon transmission, causing the left-handed rotation of the polarization plane. Conversely, D-DOPA leads to right-handed rotation, explaining their respective names. In Figure 35, L-DOPA’s spin-state electrons were mainly on the benzene ring, suggesting pre-decarboxylation fixation on the AADC enzyme similar to securing a patient during surgery. Following firm attachment, PLP facilitated decarboxylation, changing the electron spin structure as L-DOPA transformed into dopamine. The altered dopamine then dissociated from AADC for further effects, akin to post-surgery recovery, which elucidated enzyme chirality selectivity for dopamine’s chiral forms, attributing it to their distinct electron spin states, affirming the theoretical analysis in Section 3.1.

## 5. Method

With the rapid advancement of computer technology and quantum computational theory, it is now possible to conduct simulation studies of real molecular motion using computer simulation techniques. Since the discovery of electron wave properties, the resolution of human microscopes has increased dramatically, allowing for accurate representations of the structures of many biological molecules on computers. The use of probing methods for modeling significantly reduces the cost of manual modeling. The large molecular model data involved in this paper were obtained from the National Center for Biotechnology Information (NCBI). Some organic small molecules were self-modeled according to chemical formulas.

## 6. Conclusions

This paper introduces an innovative approach that integrates quantum mechanics with chemical reaction analysis, yielding a theoretical model grounded in the CISS theory. This model aptly clarifies the chiral selectivity of molecules in chemical reactions, specifically exemplified by the interaction between the AADC enzyme and dopamine molecules. The model also successfully anticipates the valence electron spin states in L-DOPA and D-DOPA molecules. Moreover, the credibility of our theory is substantiated through first-principles calculations, concurrently offering a theoretical rationale for the optical rotation of chiral substances. These discoveries not only provide a theoretical underpinning for studying the pharmacological impacts of chiral drugs but also offer insights into molecular interactions.

## Data Availability

Not applicable.

## Sample Availability

Samples of the compounds are available from the authors.
